# Manganese kinetics demonstrated a double contrast in acute but not in chronic infarction in a mouse model of myocardial ischemia reperfusion

**DOI:** 10.1186/1532-429X-13-S1-P164

**Published:** 2011-02-02

**Authors:** Bénédicte MA Delattre, Vincent Braunersreuther, Stephany Gardier, Jean-Noël Hyacinthe, Lindsey A Crowe, François Mach, Jean-Paul Vallée

**Affiliations:** 1 University of Geneva, Geneva University Hospital, Geneva, Switzerland; 2 Geneva University Hospital, Geneva, Switzerland

## 

In this study we investigated whether Manganese (Mn^2+^) wash-in kinetics can add new information regarding a myocardial infarct characterization in particular if it can differentiate an acute infarct from a chronic scar.

Manganese (Mn^2+^) is considered as a specific MRI contrast agent that enters viable cardiomyocytes through calcium pathways. Compared to extracellular Gadolinium based contrast agents, it has the potential to assess cell viability and has already shown its ability to accurately measure infarct size with late enhancement imaging. So far, only information from the wash out phase after recirculation has been used for the detection and characterization of myocardial infarct.

In this study, we used a model of 60min ischemia followed by reperfusion on C57/BL6 mice. MRI exam was performed on a clinical 3T scanner, either 24 hours (n = 10) or 8 days (n =12) after reperfusion (acute and chronic infarct respectively). Mn^2+^-induced signal intensity (SI) kinetics were measured into three distinct areas, remote, infarction and left ventricular blood pool and compared to ex vivo TTC and Masson’s trichrome.

**Figure 1:**
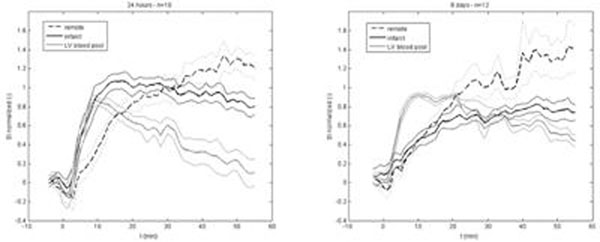
Time course of normalized SI after MnCl_2_ infusion in different areas of the myocardium (remote, infarct and left ventricular blood pool), for mice with acute (left) or chronic (right) infarction. Bold lines are mean SI and thin lines are SEM.

For both infarction types (acute and chronic) the SI kinetics showed a fast entry of the contrast agent into the blood pool followed by a slower wash-out corresponding to the recirculation phase of Mn^2+^. The SI into the remote area increased slowly from the start of the Mn^2+^ infusion to still 45 minutes later when a plateau was reached. We observed fast entry of Mn^2+^ into acute infarction followed by a recirculation phase that lead to a double contrast between infarction and “remote”, whereas the entry of Mn^2+^ into chronic infarction was slow and SI stayed lower than in “remote” (see figure 1). As a main hypothesis, extracellular space is largely enhanced in acute infarction due to cell membrane rupture (necrosis) and interstitial edema, whereas scar tissue is densely composed of collagen fibers that reduce the distribution volume of free Mn^2+^ ions.

In addition to its ability to depict accurately the infarcted area at late enhancement, Mn^2+^ is also able to discriminate acute versus chronic injury by the observation of double-contrast wash-in kinetics in a mouse model of ischemia reperfusion.

